# The role of non-steroidal anti-inflammatory drugs as adjuncts to periodontal treatment and in periodontal regeneration

**DOI:** 10.1186/s12967-023-03990-2

**Published:** 2023-02-25

**Authors:** Jianhan Ren, Melissa Rachel Fok, Yunfan Zhang, Bing Han, Yifan Lin

**Affiliations:** 1 Division of Paediatric Dentistry and Orthodontics, Faculty of Dentistry, the University of Hong Kong, Hong Kong SAR, China; 2 Division of Periodontology and Implant Dentistry, Faculty of Dentistry, the University of Hong Kong, Hong Kong SAR, China; 3 Department of Orthodontics, Cranial-Facial Growth and Development Center, Peking University School and Hospital of Stomatology, Beijing, China

**Keywords:** Non-steroidal anti-inflammatory drug, Aspirin, Periodontitis, Periodontal regeneration

## Abstract

Periodontitis is the sixth most prevalent chronic disease globally and places significant burdens on societies and economies worldwide. Behavioral modification, risk factor control, coupled with cause-related therapy have been the “gold standard” treatment for managing periodontitis. Given that host inflammatory and immunological responses play critical roles in the pathogenesis of periodontitis and impact treatment responses, several adjunctive strategies aimed at modulating host responses and improving the results of periodontal therapy and maintenance have been proposed. Of the many pharmacological host modulators, we focused on non-steroidal anti-inflammatory drugs (NSAIDs), due to their long history and extensive use in relieving inflammation and pain and reducing platelet aggregation. NSAIDs have been routinely indicated for treating rheumatic fever and osteoarthritis and utilized for the prevention of cardiovascular events. Although several efforts have been made to incorporate NSAIDs into the treatment of periodontitis, their effects on periodontal health remain poorly characterized, and concerns over the risk–benefit ratio were also raised. Moreover, there is emerging evidence highlighting the potential of NSAIDs, especially aspirin, for use in periodontal regeneration. This review summarizes and discusses the use of NSAIDs in various aspects of periodontal therapy and regeneration, demonstrating that the benefits of NSAIDs as adjuncts to conventional periodontal therapy remain controversial. More recent evidence suggests a promising role for NSAIDs in periodontal tissue engineering and regeneration.

## Introduction

Periodontal diseases are notably the most common and prevalent chronic diseases in the world [1]. They include a spectrum of inflammatory conditions encompassing gingivitis and periodontitis; the latter is a non-communicable, multifactorial chronic inflammatory infectious disease characterized by clinical attachment loss and alveolar bone destruction, which can result in tooth migration, drifting, tooth hypermobility, tooth loss, and ultimately masticatory dysfunction [2]. Periodontitis has been the major cause of tooth loss in the adult population worldwide that negatively impacts quality of life and accounts for the huge socio-economic impact and healthcare costs globally [3]. The causes of periodontitis have been extensively investigated, and its initiation and progression has been attributed to the uncontrolled accumulation of dental biofilm, which interacts with the individual susceptibility profile [4]. Genetics, tobacco and alcohol use, diabetes, obesity, poor nutrition, physical inactivity and impaired host responses have all been associated with an increased risk of periodontitis [5]. Periodontal therapy varies based on a proper diagnosis of disease severity, extent, rate of progression, risk factors, complexity of management, and interrelationship with general health [6]. According to the European Federation of Periodontology (EFP) S3 level clinical practice guideline for periodontitis [7], the fundamental cornerstones of periodontal therapy involves 4 sequential steps: Step 1—Behavior change and risk factor control; Step 2—Cause-related therapy; Step 3—Surgical interventions and Step 4—Supportive care; which are delivered in a stepwise incremental approach depending on the disease stage. The essence of the first step of therapy is both preventive and therapeutic which aims to control systemic and local risk factors. The second step of cause-related therapy targets the control of subgingival biofilm and calculus via subgingival mechanical instrumentation, namely non-surgical periodontal therapy (NSPT), and may also include the use of adjunctive physical or chemical agents, local or systemic adjunctive host-modulating agents or antimicrobials. For areas that are not responding favorably to cause-related therapy, surgical interventions may be indicated to facilitate access to root surface debridement, to regenerate or resect intra-bony or furcation periodontal defects [8, 9]. The recent advances in biological materials, therapeutic agents, and surgical techniques for regenerative periodontal therapy has enabled its success in restoring lost periodontal tissue and changing tooth prognosis [10]. While standard NSPT and supportive periodontal maintenance by means of scaling and root surface debridement remain the “gold-standard” treatment for Stage I-III periodontitis [7], there still exist patients or sites with poor response to NSPT and long-term supportive maintenance efforts that may be attributed to sustained dysbiosis, periodontopathic bacteria tissue invasion, or a non-resolving chronic inflammatory response [11]. Hence, there has been a constant pursuit for adjunctive therapies to boost the outcomes of periodontal therapy.

**Fig. 1:**
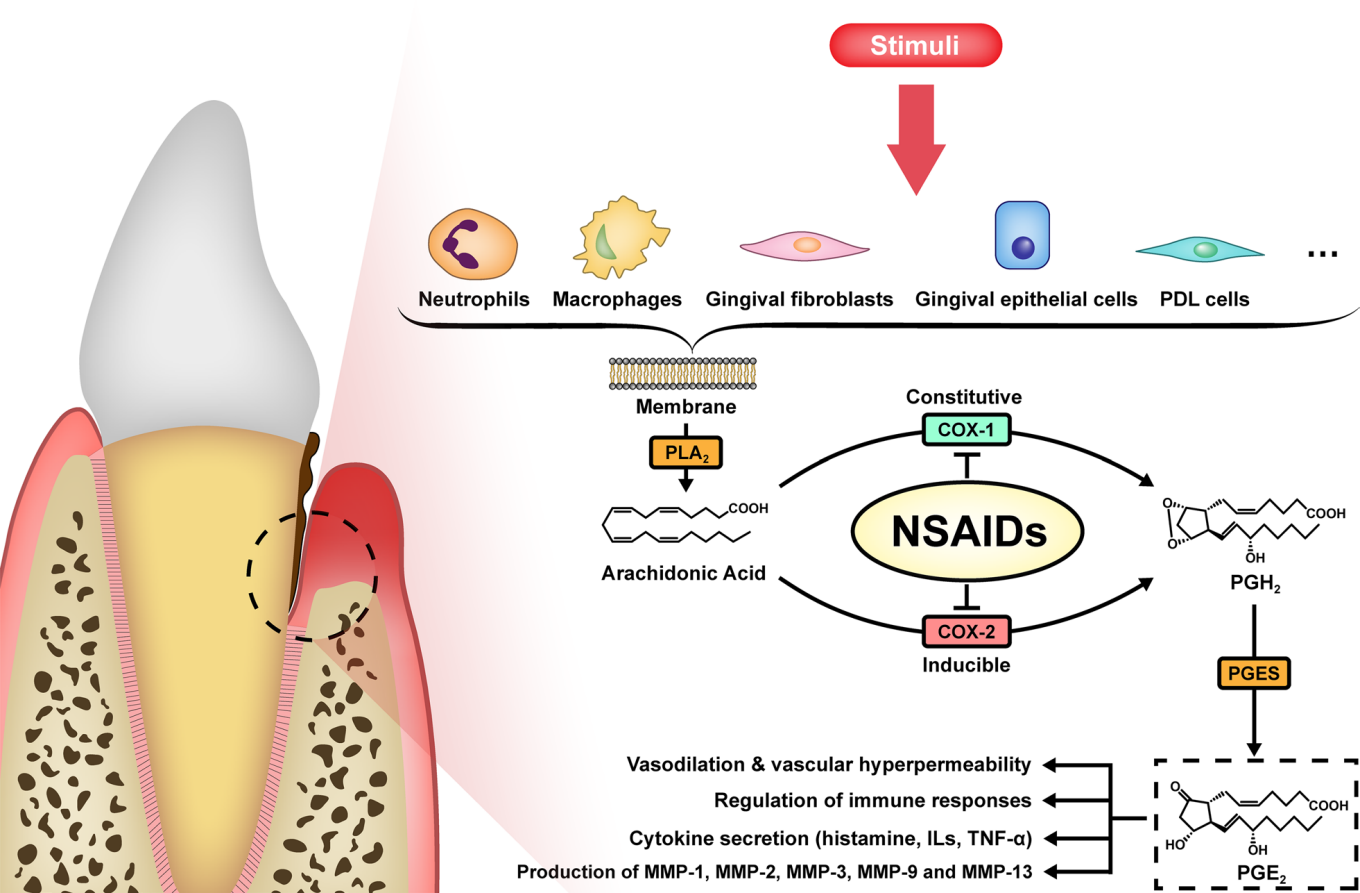
The synthesis and effects of PGE2 in periodontitis. COX-1, cyclooxygenase-1; COX-2, cyclooxygenase-2; ILs, interleukins; MMPs, matrix metalloproteinases; NSAIDs, nonsteroidal anti-inflammatory drugs; PGE synthase; PGE2, prostaglandin E2; PGES, PGH2, prostaglandin H2; PLA2, Phospholipase A2

Even though biofilms are initiators of periodontal disease, the unique individual susceptibility profile reflected in the host’s immunoinflammatory response plays an important role in periodontal tissue destruction through the local synthesis and secretion of immunological factors, and will impact the treatment responses to all four steps of periodontal therapy [12, 13]. Therefore, various host modulators have been proposed as adjuncts to conventional periodontal treatment. Among these host modulators, non-steroidal anti-inflammatory drugs (NSAIDs) are the most well-known and broadly prescribed [14]. Their pharmacological function is based on the blocking of cyclooxygenase (COX), which was discovered in the late 1980s and exists as two isoforms [15] (Fig. 1). COX-1 is the constitutive isoform and produces basal levels of prostaglandins (PGs), which control platelet activation and protect the lining of the gastrointestinal tract; COX-2 is the inducible isoform and is released in response to inflammatory stimuli such as hypoxia, interleukin (IL)-1, interferon (IFN)-γ, and tumor necrosis factor (TNF)-α, leading to swelling and pain at the inflammation site [16]. Both COX isoforms can transform arachidonic acid in the plasma membrane into PGG_2_, which is reduced to PGH_2_ by a subsequent peroxidase reaction. PGH_2_ is then converted to biologically active primary PGs: PGD_2_, PGE_2_, PGF_2_ $\alpha$, PGI_2_, and thromboxane A_2_ (TxA_2_) [15].

NSAIDs are classified into four categories based on their affinity and/or selectivity for COX isoenzymes (Table 1). The first category comprises compounds with potential to produce complete inhibition of both COX-1 and COX-2 and is exemplified by aspirin, indomethacin, diclofenac, naproxen, and ibuprofen. The second category of compounds consists of preferential COX-2 inhibitors (5- to 50-fold selectivity) and includes meloxicam, celecoxib, nimesulide, and etodolac. The third category comprises compounds with strong selectivity (> 50-fold) for COX-2, such as diisopropyl fluorophosphate, L-745337, rofecoxib, and NS398. The fourth category of compounds has low affinities for both COX-1 and COX-2 and comprises compounds such as sulfasalazine, sodium salicylate, and nabumetone [17, 18]. Notably, aspirin is unique in that it is an irreversible inhibitor of COXs, whereas all of the other NSAIDs exhibit some degree of reversible inhibition [19].

**Table 1:** Classification of NSAIDs according to their selectivity for COX isoenzymes

Category	Properties	Examples
1	Inhibitors of COX-1 and COX-2	indomethacin, aspirin, diclofenac, naproxen, ibuprofen
2	5- to 50-fold COX-2 selective	etodolac, meloxicam, celecoxib, nimesulide
3	> 50-fold COX-2 selective	diisopropyl fluorophosphate, L-745337, rofecoxib, NS398
4	Weak inhibitors of COX-1 and COX-2	5-Aminosalicylic acid, nabumetone, sodium salicylate, sulfasalazine

NSAIDs can inhibit PG synthesis and consequently exert analgesic, anti-pyretic, and anti-inflammatory effects. Therefore, they are indicated to relieve pain and fever and treat inflammation in rheumatic, osteoarthritic, and other diseases. In addition, they can be used to prevent cardiovascular events, premature labor, and perturb the microenvironments of tumors [20, 21]. However, some nonspecific cytotoxic effects of NSAIDs can be detrimental to the human body; such complications should not be ignored [22].

In this review, we summarize laboratory and clinical evidence for the utility of NSAIDs in the conventional treatment of periodontitis, which includes both surgical and non-surgical periodontal treatment, but without regenerative approaches, and in periodontal tissue engineering and regeneration. In the first part of the review, we discuss the role of PGs in periodontitis and the administration of NSAIDs during cause-related non-surgical or surgical therapies. In the second part of the review, we describe the combination of NSAIDs with current periodontal regeneration techniques.

## NSAIDs as adjuncts to conventional periodontal treatment

### The role of PGs in periodontitis

#### PGs in the pathogenesis of periodontitis

Arachidonic acid metabolites have been implicated in a variety of inflammatory conditions such as rheumatoid arthritis, psoriasis, diabetes mellitus, obesity, and periodontitis [13, 23–25]. Among the subfamilies of arachidonic acid metabolites, PGs are the most critical inflammatory mediators of periodontitis. They regulate local inflammation in both autocrine and paracrine manners, with impacts on vasodilation, vascular permeability, edema, and pain [26]. PGE_2_, one of the most abundant PGs in periodontal tissue, plays a prominent role in pathogenesis. In periodontal tissue, PGE_2_ has been shown to be produced by neutrophils, macrophages, periodontal ligament cells, gingival fibroblast cells, gingival epithelial cells, osteoblasts, and cementoblasts [27–32]. PGE_2_ is mainly known for its pro-inflammatory effects. It causes vasodilation and vascular hyperpermeability, facilitating the leakage of immune cells and plasma components into extracellular space and leading to local inflammation [33].

Recently, lymphocyte responses have been recognized as being involved in the onset of periodontal diseases; PGE_2_ has been shown to directly stimulate T cell activation and the production of various cytokines. Yao et al. reported that PGE_2_ promotes Th1 cell differentiation and Th17 cell expansion both in vitro and in vivo [34, 35]. Th1 cells secrete IFN-γ and other cytokines, activating immune cells such as macrophages, dendritic cells, and B cells, which play a critical role in defending the body against infectious diseases such as periodontitis [36]. In addition, PGE_2_ stimulates the production of IL-17A by Th17 cells, and that IL-17A can in turn increase the expression of PGE_2_ in osteoblasts [37, 38], indicating a magnification of the effects of PGE_2_ at the site of periodontitis.

A growing body of clinical evidence also suggests the relationship between PGE_2_ and periodontitis. Goodson et al. first reported a tenfold increase in PGE_2_ concentrations in periodontally compromised tissues compared with healthy tissues [39]. Varying concentrations of PGE_2_ have been observed in gingival crevicular fluid, which were later shown to be correlated with the severity of periodontal inflammation [40, 41]. High concentrations of PGE_2_ observed in the saliva of patients with severe periodontitis and high PPDs [42].

#### PGE_2_ and periodontal tissue destruction

The destruction of both soft and hard tissues in periodontitis is largely attributable to the inflammatory host responses to pathogens [43]. Of the various cytokines and growth factors secreted at the sites of periodontitis, matrix metalloproteinases (MMPs) is one of the most vital contributors to soft tissue destruction by degrading collagen within the gingiva and periodontal ligament [44]. Yen et al. [45] and Khan et al. [46] demonstrated that PGE_2_ directly promotes the expression of MMP-9 in dendritic cells and macrophages; other MMPs such as MMP-1, MMP-2, MMP-3, and MMP-13 have also been shown to be regulated by PGE_2_ [32, 47–49]. Furthermore, PGE_2_ may also indirectly activate MMPs by inducing the production of pro-inflammatory cytokines that regulate MMPs, such as IL-1β, IL-6, and IL-17, during the progression of periodontitis [50, 51].

Regarding hard tissue destruction, early in vitro and in vivo studies have shown the effects of PGE_1_ and PGE_2_ on bone resorption [52, 53]. Studies have demonstrated the direct role of PGE2 in bone resorption by showing that PGE2 can bind to its receptors (EPs) on osteoclasts, thereby stimulating osteoclastic activity [54, 55]. Another mechanism by which PGE_2_ induces osteoclastogenesis is cellular communication between osteoclasts and osteoblastic cells through the receptor-activator of nuclear factor-κB ligand (RANKL)/receptor-activator of nuclear factor-κB (RANK)/osteoprotegerin (OPG) pathway. It has been shown that PGE_2_ upregulates the expression of RANKL, which binds to its receptor RANK on osteoclastic precursors to facilitate differentiation and downregulate the expression of OPG in mesenchymal lineage cells [56]. In periodontal tissue, PGE_2_ exposure has been reported to inhibit OPG expression in both periodontal ligament cells and osteoblasts while selectively promoting RANKL expression [57]. Collectively, this overwhelming body of evidence has confirmed the participation of PGE_2_ in the pathogenesis of periodontal diseases and its destructive impact on periodontal tissue.

### Application of NSAIDs in non-clinical studies

Given the critical role of PGs, especially PGE_2_, in the progression of periodontal diseases, therapeutic strategies that use NSAIDs in periodontitis have been explored. The first investigation in this field was conducted 2 years after the mechanisms of NSAIDs were elucidated in 1971 [58]. In an in vitro study, Goldhaber et al. [59] reported that blocking PG synthesis by indomethacin significantly alleviated alveolar bone resorption caused by gingival cytokines in a dose-dependent manner, indicating that it acted on local inflammatory responses. To further examine the effects of indomethacin in vivo, Nyman et al. [60] established a ligature-induced periodontitis model in beagle dogs. The dogs were administered either no drug or indomethacin at 1 mg/kg orally for 29 days. Periodontal tissue was then biopsied and analyzed for the progression of inflammatory reactions and alveolar bone resorption. The results demonstrated that indomethacin delayed the initiation and reduced the severity of ligature-induced periodontitis and suppressed bone resorption. The findings were further verified by another study in squirrel monkeys [61].

Flurbiprofen is another NSAID that has been frequently explored in the context of therapies for periodontitis. Williams et al. [62] explored the effects of flurbiprofen on periodontitis in beagles by administering it to the dogs for 12 months. The bone loss rates of the treated groups largely decreased after drug administration compared with the baseline, indicating This that flurbiprofen could be used as an adjunct to periodontal surgical treatment. A subsequent study with the same animal model was conducted to explore whether the therapeutic effects of flurbiprofen could be sustained after terminating drug administration [63]. The rate of bone loss decreased over the course of pharmaceutical treatment, but returned to baseline at the sixth month after discontinuing treatment. Interestingly, despite the profound reduction in alveolar bone loss by flurbiprofen, gingival inflammation was not affected during the whole process.

Williams et al. [64] further investigated the effects of ibuprofen, an analogue of flurbiprofen, on periodontitis in beagle dogs. They employed a polymer matrix for the sustained release of ibuprofen. Although both normal and sustained release of the drug inhibited alveolar bone loss, sustained release was more effective than normal administration. Furthermore, the dogs in the sustained release group lost fewer teeth during the 7-month treatment period than those in other groups. These results highlight a more potent way of continuously releasing and obtaining a consistently high blood concentration of the drug. The authors also demonstrated the preventive effect of another NSAID, naproxen, against alveolar bone loss in beagle dogs, although this effect was attenuated after long-term use [65].

### Application of NSAIDs in clinical studies

Significant efforts have been made to evaluate the application of NSAIDs in the progression of periodontal diseases in humans. Some earlier studies have shown that NSAIDs may have a therapeutic role in the process. Jeffcoat et al. [66] recruited 15 patients with active moderate-to-severe periodontitis and administered 50 mg b.i.d. flurbiprofen or placebo for 2 months. Radiography showed significantly less bone loss during the 2-month study period in the flurbiprofen-treated patients compared with the placebo-treated patients. More tooth sites with bone loss were also detected in the placebo group than in the flurbiprofen-treated group. In a 44-patient study conducted by Williams et al. [67], periodontitis patients were prescribed 50 mg b.i.d. flurbiprofen or placebo alongside NSPT for 2 years. Compared with the control group, the flurbiprofen-treated patients reported a lower rate of bone loss and less bleeding at probing spots after 1 year of therapy.

However, more recent clinical studies have revealed conflicting results regarding the treatment of periodontitis with NSAIDs. Haffajee et al. [68] examined the effects of three adjunct medications in periodontal treatment. The patients were given ibuprofen (400 mg t.i.d.), antibiotics (Augmentin™ [amoxycillin with clavulanic acid] or tetracycline), or a placebo, during a 1-month period of active periodontal treatment. Ibuprofen and placebo treatment exhibited similar decreases in PPD and gains or losses of clinical attachment level (CAL), which were inferior to the effects of treatment with either of the two antibiotics. A similar study on the adjunctive benefits of flurbiprofen administered at 200 mg q.d. during 10 days of periodontal therapy was conducted on periodontitis patients with different smoking statuses [69]. After periodontal therapy, a reduction in the plaque and gingival index was observed in all groups, while the PPD and CAL remained unchanged. However, NSAID treatment had no impact on either the clinical or biological parameters. Notably, the medicine was administered only for a short period of periodontal treatment in these studies. This was different from previous studies, wherein the effects of NSAIDs became apparent only after months of application [67].

A longer treatment duration with NSAIDs, in conjunction with conventional periodontal therapy, has been shown to improve periodontal outcomes. A 6-week administration of naproxen improved clinical parameters such as the gingival index, plaque index, and PPD, but no difference was observed in immunological parameters compared with placebo-treated patients [70]. Yen et al. [71] investigated a more extended application of celecoxib, a COX-2 inhibitor, for 12 months. They systematically prescribed 131 patients with chronic periodontitis either celecoxib or placebo combined with SRP. Compared with patients in the placebo group, patients administered celecoxib showed more prominent PPD reduction and CAL gains, especially in moderate-to-severe pockets. Furthermore, celecoxib-treated patients showed more sites with attachment gains and fewer sites with attachment loss than those in the placebo group. Bleeding on probing also improved in celecoxib-treated patients. Oduncuoglu et al.[72] employed a cyclic regimen with 50 mg diclofenac potassium for 2 months followed by 2 months of washout and another 2 months of prescription, and their group reported significant reduction in PPD at 6 months. However, regarding the topical application of NSAIDs, the current findings are inconclusive. The use of a 1% flurbiprofen toothpaste twice a day for 12 months did not improve PPD reduction compared to a placebo toothpaste [73]. In another randomized controlled trial, supragingival daily irrigation with 300 ml of water immediately followed by 200 ml of buffered 0.3% acetylsalicylic acid did not improve PPD at 6 months compared to irrigation with water alone [74]. Due to the conflicting results so far, future studies are still needed to elucidate the efficacy of the adjunctive use of NSAIDs to subgingival instrumentation in different conditions.

### A focus on aspirin

Aspirin is an ancient analgesic and anti-pyretic drug. The history of using salicylic acid, the major metabolite of aspirin, can be traced back to 3,500 years ago, when willow bark was utilized as a traditional medicine by ancient Sumerians and Egyptians [75]. In recent years, researchers have explored whether aspirin can also be used to in the treatment of periodontitis. Coimbra et al. [76] investigated the effects of aspirin on ligature-induced periodontitis in rats. The animals in the experimental group were administered aspirin intragastrically at 30 mg/kg body weight for 15 days. Aspirin significantly reduced the level of inflammation when assessed by histometric analysis and in terms of inflammatory factors such as TxA_2_, TNF-α, and IL-6.

In addition to its anti-inflammatory effect at high doses via the inhibition of COX-1/2, aspirin may also contribute to inflammation relief through other mechanisms. Lipoxin (LX) is an arachidonic acid metabolite synthesized by lipoxygenases (LOs). Aspirin acetylates COX-2 to generate the LX epimers 15-epi-LXA4 and 15-epi-LXB4 in the presence of 5-LO. These epimers, termed aspirin-triggered lipoxins (ATLs), can better alleviate inflammation than native LXs and have been used to treat diseases such as asthma, peritonitis, sepsis, and cardiovascular diseases [77, 78]. Serhan et al. [77] showed that the topical application of ATLs drastically inhibits inflammatory infiltration and alveolar bone loss in a rabbit periodontitis model. Other studies have documented that aspirin resulted in the synthesis of aspirin-triggered resolvins and aspirin-triggered protectins, which are anti-inflammatory molecules, along with omega-3 polyunsaturated fatty acids (PUFAs) [79, 80]. El-Sharkaway et al. [81] reported significant PPD improvements and reduced number of sites requiring additional therapy with the intervention of 3 g omega-3 PUFA with 81 mg aspirin daily for 6 months. A recent study by Castro Dos Santos et al. [82] showed that aspirin, administered at a low dose of 100 mg/day in conjunction with 3 g of fish oil for 2 months, improved both clinical and immunological parameters in patients with periodontitis and type 2 diabetes. Specifically, in patients taking medication after conventional periodontal treatment, aspirin in combination with omega-3 PUFA significantly improved clinical attachment level gains, reduced IL-6, IL-8, and IFN-γ in gingival crevicular fluid, and decreased HbA1c concentrations in peripheral blood.

In clinical settings, aspirin has long been employed for the treatment of various diseases and conditions such as headache, osteoarthritis, rheumatic diseases, cardiovascular diseases, and even cancers because of its versatile pharmacologic activities of pain relief, anti-inflammation, and anti-platelet function [83–85]. Therefore, several observational studies have been carried out to investigate its effects in subjects with a history of aspirin intake in terms of periodontal parameters. Feldman et al. [86] demonstrated that arthritic patients with daily consumption of aspirin and indomethacin presented significantly fewer sites with ≥ 10% bone loss than healthy individuals, highlighting the possible protective effect of aspirin on periodontal tissue. A similar finding was reported in a later study, suggesting that low-dose aspirin may reduce the risk of clinical attachment loss [87]. However, a nationally representative report from the United States involving 2,335 subjects demonstrated that aspirin users and non-users did not differ significantly in terms of PPD and clinical attachment loss, suggesting that low-dose aspirin use is not associated with the reduced prevalence of periodontitis [88]. It is also critical to understand that daily aspirin exposure has been shown to increase the appearance of bleeding on probing that could impact on diagnosis and treatment planning for periodontitis [89, 90].

Smokers are prone to periodontal disease and tend to have inferior responses to non-surgical and surgical periodontal treatment compared to non-smokers [91–93]. A cross-sectional study of non-smokers and ex-smokers who took low-dose aspirin (≤ 300 mg/day) for at least 2 years showed that aspirin takers had less attachment loss (by 0.3 mm) than non-aspirin takers, regardless of smoking status [94]. Another recent randomized controlled pilot study [95] explored the effects of long-term aspirin use as an adjunct to periodontal therapy in smokers. In conjunction with SRP, patients administered 325 mg/day of aspirin for 12 months showed better improvements in clinical parameters such as PPD and CAL than those in the placebo group, with no adverse effects.

## NSAIDs in periodontal tissue engineering and regeneration

Non-surgical and access flap periodontal surgeries in anatomically challenging areas are effective procedures for mechanically removing biofilms and thereby relieve local inflammation in patients with periodontal diseases. These techniques aim to remove infectious substances, halt disease progression, and restore periodontal health; however, healing is mainly by repair and not regeneration of lost periodontal tissues [96]. To regenerate new supportive apparatus for teeth, strategies such as, minimally invasive surgical techniques in periodontal regeneration [97, 98] in combination with biologics, guided tissue regeneration (GTR), bone replacement grafts, and periodontal tissue engineering have been developed [99]. GTR involves placing an absorbable or non-absorbable membrane above the area to be restored. This prevents the undesired migration of epithelial cells and gingival fibroblasts and promotes the growth of bone progenitor cells [100]. The benefits of GTR have been adequately discussed in previous reviews, such as decreased PPD, increased gains in attainment level, and the formation of new bone [101, 102].

Currently, bone replacement grafts, biologics such as amelogenins and various biomaterials can be utilized either by themselves or as scaffolds in conjunction with different surgical techniques. Bone replacement grafts use osteoinductive and osteoconductive bone graft materials to fill the bone defect areas, with the aim of regenerating the most important component of supportive tissue, the alveolar bone [103]. Common graft materials are autologous bone, bovine xenograft, and demineralized freeze-dried bone allograft (DFDBA) [104–106]. Several efforts have been dedicated to exploring novel biomaterials in recent decades [107]. Biomaterials are normally used as scaffolds in tissue engineering to facilitate cell attachment, tissue formation, and mechanical support. Given the complexity of the periodontium, materials such as hydroxyapatite (HA), biphasic calcium phosphate, and tricalcium phosphate (TCP) have been used to reconstruct bone tissue [108]. Some innovative biomaterials that mimic the structure and characteristics of the cementum, periodontal ligament, and alveolar bone have also been used to restore naturally structured periodontal tissues [109].

### Anti-inflammatory effects of NSAIDs on periodontal regeneration

Despite the added benefits of periodontal regeneration procedures as compared with access flap procedure in terms of gains in clinical attachment, there is a significant inter-center variability in the outcomes [110] due to the implanted biomaterials-triggered inflammatory responses [111] or systemic inflammatory conditions such as diabetes mellitus. Therefore, anti-inflammatory biomaterials design strategies, such as modification of surface chemistry, topography, and biomolecules, have been developed to reduce the adverse immunological responses in periodontal regeneration [112].

The incorporation of NSAIDs in biomaterial synthesis has been extensively studied (summarized in the Table 2). Veronese et al. [113] manufactured biodegradable polyphosphazene membranes and microspheres for periodontal regeneration. These materials were loaded with naproxen and had a release rate that yielded therapeutically adequate blood concentrations. However, the biological effects of this biomaterial were not investigated. The incorporation of salicylic acid into poly(anhydride-ester) was shown to relieve inflammation around implants [114], and promote bone regeneration more effectively than the bone graft alone in a critical-size bone defect model in rats [115]. The effects of ibuprofen were also explored on a polycaprolactone (PCL) membrane. An in vivo study using a mouse periodontitis model demonstrated that the combination of ibuprofen and PCL membrane prevented clinical attachment loss and inhibited osteoclastogenesis, thereby improving periodontal regeneration [116].

**Table 2:** NSAID application in periodontal tissue engineering and regeneration

Year	Drug	Material	Study type	In vivo design	Outcome	Mechanism	Ref
2007	SA/diflunisal	PAE	in vivo	Rat calvarial bone defect model	Relieved inflammation around implants	Anti-inflammation	[114]
2013	SA	PAE	in vivo	Rat mandibular bone defect model	Enhanced bone regeneration	Anti-inflammation	[115]
2018	Ibuprofen	PCL	in vitro & in vivo	Mouse periodontitis model	Improved CAL and inhibited bone destruction	Anti-inflammation; inhibited migration of ECs and FBs	[116]
2018	Diclofenac	Alginate/CS hydrogel fibers	in vitro	–	Enhanced bone osteogenesis and relieved inflammation	Anti-inflammation	[120]
2011	Aspirin + omega-3 polyunsaturated fatty acids	DFDBA	clinical	Randomized controlled trial (a total of 40 patients)	Improved CAL, probing depth, gingival index, and gingival bleeding index at 6-month follow-up	Anti-inflammation	[117]
2011	Aspirin	Heprasil/Gelin-S hydrogel	in vivo	Rat calvarial bone defect model	Enhanced bone regeneration	Modulation of T cells	[126]
2017	Aspirin	HA/TCP	in vitro & in vivo	Rat mandibular bone defect model	Enhanced bone regeneration	Inhibited M1 polarization of macrophages	[121]
2018	Aspirin	Bio-Oss^®^	in vitro & in vivo	Rat calvarial bone defect model	Enhanced bone regeneration	Improved osteogenic differentiation of DPSCs	[129]
2019	Aspirin	Liposomes/PCL	in vitro & in vivo	Subcutaneous implantation in nude mice	Enhanced bone regeneration	Anti-inflammation; improved osteogenic differentiation of MSCs	[135]
2019	Aspirin + EPO	CS/b-GP/gelatin hydrogel	in vivo	Rat periodontitis model	Improved CAL and inhibited bone destruction	Anti-inflammation	[137]
2019	Aspirin	HA/TCP & hydrogel	in vitro & in vivo	Rat mandibular bone defect model	Enhanced bone regeneration	Inhibited osteoclastogenic differentiation of DCs	[139]
2020	Aspirin	Sr-α-CSH/n-HA	in vitro & in vivo	Rat tibial bone defect model	Enhanced bone regeneration	Improved osteogenic differentiation, proliferation, and migration of BMSCs	[134]
2022	Aspirin	Tetra-PEG-CS composite gel	in vitro & in vivo	Rat calvarial bone defect model	Enhanced bone regeneration	Enhanced M2 polarization of macrophages; improved osteogenic differentiation and proliferation of PDLSCs	[136]

CAL, clinical attachment level; CS, chitosan; DCs, dendric cells; DFDBA, demineralized freeze-dried bone allograft; DPSCs, dental pulp stem cells; ECs, epithelial cells; FBs, fibroblasts; HA, hydroxyapatite; MSCs, mesenchymal stem cells; PAE, poly(anhydride-ester); PCL, polycaprolactone; PDLSCs, periodontal ligament stem cells; PEG, poly(ethylene glycol); SA, salicylic acid; Sr-α-CSH/n-HA, Strontium (Sr)-containing α-hemihydrate calcium sulfate/nano-hydroxyapatite; TCP, tricalcium phosphate

The combination of omega-3 PUFA and low-dose aspirin was found to be effective as an adjunct to regenerative periodontal surgery. Elkhouli et al. [117] demonstrated that DFDBA implantation in conjunction with omega-3 PUFA and low-dose aspirin administration for 6 months resulted in greater PPD reduction and increased gains in CAL compared with DFDBA alone. IL-1β and IL-10 concentrations in gingival crevicular fluid were also downregulated by the administration of omega-3 PUFA and low-dose aspirin.

Of the various cell types involved in bone remodeling, macrophages are of great importance as they can switch to either the pro-inflammatory M1 or anti-inflammatory M2 phenotype and thus secrete different cytokines to regulate bone formation [118]. The typical cytokines produced by M1 macrophages are inducible nitric oxide synthase (iNOS), IL-1β, and TNF-α, which are associated with inflammatory responses. M2 macrophages secrete cytokines such as IL-4, IL-10, and TGF-β and promote cell proliferation and tissue regeneration [119]. As excessive inflammation hinders bone regeneration, some strategies have been developed to regulate the biology of macrophages. Lin et al. [120] established a natural polymer system for the sustained release of diclofenac, an NSAID. They embedded diclofenac in a porous alginate hydrogel scaffold and coated it with a layer of chitosan hydrogel to slow the release of diclofenac. When co-cultured with LPS-stimulated macrophages, this biomaterial significantly reduced iNOS, IL-6, and TNF-α secretion from the cells, thus suppressing inflammation. Furthermore, the direct treatment of macrophages with aspirin was reported to inhibit their LPS-stimulated phenotypes [121]. Mechanistically, aspirin was shown to downregulate the expression of iNOS and TNF-α via the COX_2_/PGE_2_/EP_2_/nuclear factor (NF)-κB and the inhibitor of NF-κB kinase (IκK) /inhibitor of NF-κB (IκB)/NF-κB signaling pathways. Further in vivo studies confirmed that aspirin inhibits the infiltration of LPS-induced iNOS^+^ macrophages and thereby promotes the regeneration of periodontal alveolar bone [121].

However, in terms of bone fracture healing, NSAIDs have typically been associated with delayed healing [122]. The inhibition of cyclooxygenase is documented to induce fracture non-unions and incomplete unions [123]. One possible explanation for their different effects on bone fracture healing and periodontal bone regeneration might be due to their different biological microenvironments. During periodontal regeneration, residual bacteria, newly formed biofilm, and the foreign body response to implanted biomaterials may intensify inflammation to a degree greater than that in closed fracture healing where COX-2 inhibitors can act to dampen the inflammation to a level more compatible for regeneration to occur, as opposed to closed bone fracture healing where the physiological level of naturally occurring inflammation and cyclooxygenase level might be more optimal for angiogenesis, osteogenesis and healing to occur [124]. Since it has been established that excessive inflammation impairs tissue regeneration [125], the involvement of inflammatory inhibitors may benefit periodontal regeneration. One study showed that a BMSC-based scaffold failed to regenerate tissue due to the elevated secretion of the inflammatory factors IFN-γ and TNF-α by T cells; relieving this inflammation by aspirin improved the regenerative progress [126].

### Effects of aspirin on periodontal regeneration

**Fig. 2:**
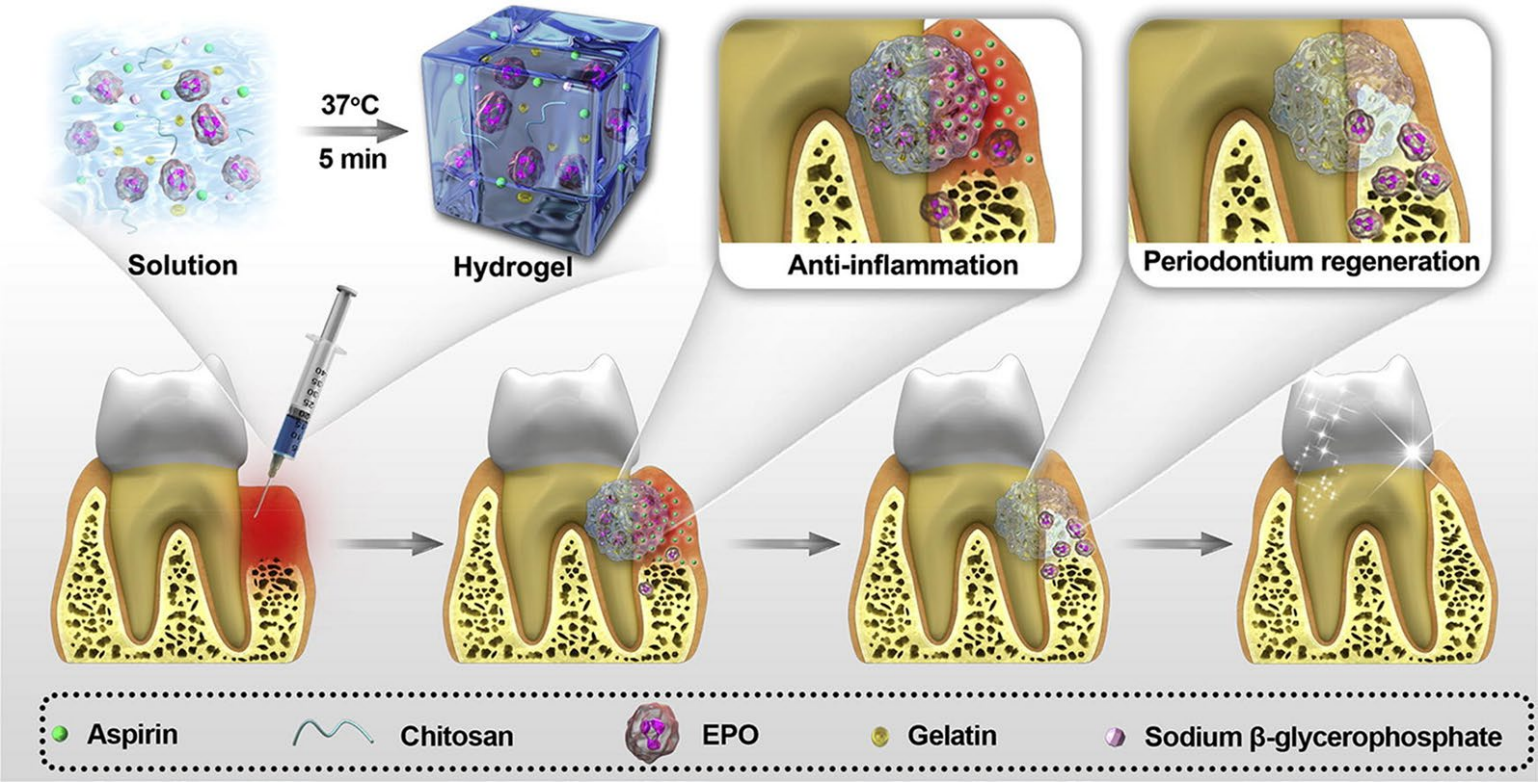
A representative application of aspirin-loaded biomaterials in periodontal regeneration. Reproduced with permission from reference [137]. Copyright 2019 Elsevier

The distinct effect of aspirin on bone metabolism, supplementary to its anti-inflammatory effect, has increasingly attracted the attention of researchers. Inspired by an epidemiological study that indicated some beneficial effects of aspirin on bone mineral density [127], Yamaza et al. [128] investigated the association between aspirin and bone homeostasis. In an ovariectomy-induced osteoporosis model in mice, the systemic delivery of aspirin significantly improved trabecular and cortical bone density. They established that aspirin blocked Fas ligand-induced BMSC apoptosis and promoted the osteogenesis of BMSCs by increasing telomerase activity. As BMSCs are the most important cellular reservoirs for bone formation and the most commonly used seed cells in bone tissue engineering, the effects of aspirin on BMSCs may impact bone regeneration. Further studies demonstrated that aspirin could improve the osteogenic differentiation of dental pulp and periodontal ligament stem cells [129, 130]. The adipogenesis of BMSCs was also inhibited by aspirin via histone acetylation [131]. Adipogenesis and osteogenesis are generally two sides of cell fate determination, with factors inducing adipogenesis often inhibiting osteogenesis and vice versa [132]. Furthermore, aspirin has been reported to enhance cell migration and stem cell homing [133]. Together, these studies suggest a pro-osteogenic effect of aspirin that can potentially benefit bone formation during periodontal regeneration. Figure 2 shows a representative utilization of aspirin in periodontal regeneration.

Strontium (Sr)-containing α-hemihydrate calcium sulfate/nano-hydroxyapatite (Sr-α-CSH/n-HA) is a biocompatible, osteoinductive, and osteoconductive biomaterial with weak compressive strength. Jiang et al. [134] added aspirin to balance the concentration of Sr and develop a material suitable for bone regeneration. In a critical-sized tibial bone defect model in rats, the increasing amount of aspirin in the material enhanced the number of cells undergoing osteogenic differentiation and inhibited osteoclastogenesis. Micro-computed tomography images revealed that the overall bone formation was significantly improved by aspirin, which also inhibited osteoclastogenesis around the defect. In vitro experiments showed that BMSCs co-cultured with the aspirin-loaded Sr-α-CSH/n-HA scaffold exhibited better osteogenic ability, proliferation, and migration ability than the scaffold without aspirin.

Liposomes, as a novel delivery system, can directly deliver active molecules to the site of action, reduce systematic toxicity, and achieve sustained release. Aspirin-laden liposomes on PCL-based composite scaffolds have been shown to significantly promote mineralization and osteogenic gene expression and inhibit osteoclastogenesis and the expression of inflammatory factors such as TNF-α and IFN-γ [135]. The subcutaneous implantation of scaffolds containing aspirin in nude mice revealed its elevated potential for bone regeneration.

Hydrogel, due to its excellent biocompatibility and biodegradability, can also be used as a scaffold for aspirin delivery. Tetra-armed poly(ethylene glycol)-chitosan hydrogels encapsulating aspirin were shown to possess favorable physicochemical properties and suitable pore structure for periodontal regeneration [136]. Aspirin was released from these scaffolds in a sustained manner and promoted osteogenesis in vitro. In a critical-size defect model in mice, hydrogels loaded with aspirin resulted in a significantly higher volume of newly formed bone than hydrogels without aspirin. The underlying mechanism was the facilitation of M2 polarization by aspirin. Chitosan β-sodium glycerophosphate/gelatin injectable hydrogels combined with aspirin and erythropoietin (EPO) were established as a controlled-release system to regenerate periodontal tissues [137]. When injected subcutaneously into nude mice, these hydrogels in conjunction with aspirin showed strong anti-inflammatory effects coupled with a reduction in COX-2 and MMP-9 expression. In a periodontitis model, the use of aspirin-loaded hydrogels also yielded better gains in the CAL and greater bone regeneration than the use of hydrogels alone, with the combination of aspirin and EPO showing the most robust effects.

Aspirin may also enhance bone formation by modifying osteoclastogenesis. Zeng et al. [138] assessed the effects of aspirin on osteoclastogenic differentiation in the mouse macrophage cell line RAW264.7. Aspirin significantly reduced the number of differentiated osteoclasts and suppressed the mitogen-activated protein kinase (MAPK) and NF-κB signaling pathways in RANKL-treated cells. Another study showed that aspirin could inhibit the osteoclastogenesis of dendritic cells [139]. In this study, loading aspirin onto HA/TCP hydrogels also improved bone regeneration in a rat mandibular defect model.

## Conclusions and future perspectives

Although several studies have investigated the effects of NSAIDs on the treatment of periodontitis, either alone or as adjuncts to non-surgical or surgical periodontal treatments, it remains challenging to draw a definitive conclusion on their efficacy. This is exacerbated by the fact that conventional periodontal therapies have proven safe and efficacious in resolving inflammation and halting tissue destruction [140, 141], making it unnecessary to use NSAIDs as adjunctive therapy for most conditions [7]. Furthermore, NSAIDs have long-term adverse effects that cannot be ignored; non-selective NSAIDs can cause gastrointestinal bleeding, renal effects, hypertension, hepatic injury, and headache, and COX-2 inhibitors have similar adverse cardiovascular and renal effects [142, 143]. Moreover, aspirin exerts anti-platelet effects even at low doses, as it inhibits thromboxane synthesis, which increases the risk of bleeding [144]. This may increase periodontal bleeding in patients with or without periodontal diseases [89, 145, 146]. Therefore, considering the costs and benefits, systemic or local use of NSAIDs as an adjunct to conventional periodontal treatment is not recommended by the EFP [7].

Notwithstanding the limitations, recent preclinical and clinical research on host modulation utilizing NSAIDs, especially aspirin, in combination with omega-3 PUFA for NSPT and alone in periodontal regeneration has yielded promising outcomes. These promising preliminary results signal their potential use as adjunctive treatment in selective patient groups with high susceptibility risk profile or poor response. Studies on the mechanism of action of aspirin have shown that, in addition to inhibiting PG synthesis, it activates various signaling pathways [147–149]. They have also documented its role in promoting osteogenesis, inhibiting adipogenesis, attenuating osteoclastogenesis, modulating macrophage polarization, and inducing cell migration and stem cell homing, all of which support periodontal regeneration. However, some of the results must be interpreted with caution as many of these preclinical studies have used critical-size defect models of the tibia and cranium in animals. Given the position of defects, local infection, and inflammation level, it remains to be determined whether the results from these models can precisely reflect the chronic inflammation condition in the periodontium. Well-designed, high quality randomized controlled trials investigating the adjunctive use of NSAIDs in periodontal therapy alone or in combination are also needed to further substantiate their additional benefits, determine dosing regimen, and establish safety.

The timing of administering NSAIDs also warrants consideration. Tissue healing comprises two inflammatory phases, the initiation and resolution of acute inflammation, characterized by immune cell infiltration, cellular debris clearance, and the production of cytokines and growth factors that facilitate tissue healing [122]. Disturbing either of these phases may delay healing [150]. Moreover, it has been shown that pro-inflammatory signals, when delivered at the early inflammatory stage, can aid tissue repair [151]. Therefore, the controlled release of anti-inflammatory drugs in the resolution phase, rather than the initiation phase, may boost tissue regeneration [115]. Smart biomaterials that can sense, respond, and adapt to stimuli have attracted increasing attention [152]. It is thus possible that a combination of anti-inflammatory agents and smart biomaterials will generate a precisely controlled microenvironment that can maximize the benefits and potential for periodontal regeneration in the future.

## Data Availability

Not applicable.

## Abbreviations

ATLs: Aspirin-triggered lipoxins
BMSCs: Bone marrow stem cells
CAL: Clinical attachment level
COX: Cyclooxygenase
CS: Chitosan
DCs: Dendric cells
DFDBA: Demineralized freeze-dried bone allograft
DPSCs: Dental pulp stem cells
ECs: Epithelial cells
EFP: European Federation of Periodontology
EPO: Erythropoietin
EPs: PGE_2_ receptors
FBs: Fibroblasts
GCF: Gingival crevicular fluid
GTR: Guided tissue regeneration
HA: Hydroxyapatite
IFN: Interferon
IL: Interleukin
iNOS: Inducible nitric oxide synthase
LOs: Lipoxygenases
LPS: Lipopolysaccharide
LX: Lipoxin
MAPK: Mitogen-activated protein kinase
MMPs: Matrix metalloproteinases
MSCs: Mesenchymal stem cells
NSAIDs: Non-steroidal anti-inflammatory drugs
NSPT: Non-surgical periodontal therapy
OPG: Osteoprotegerin
PAE: Poly(anhydride-ester)
PCL: Polycaprolactone
PDLSCs: Periodontal ligament stem cells
PEG: Poly(ethylene glycol)
PGs: Prostaglandins
PPD: Probing pocket depth
PUFAs: Polyunsaturated fatty acids
RANK: Receptor-activator of nuclear factor-κB
RANKL: Receptor-activator of nuclear factor-κB ligand
SA: Salicylic acid
Sr-α-CSH: Strontium (Sr)-containing α-hemihydrate calcium sulfate
TCP: Tricalcium phosphate
TNF: Tumor necrosis factor
TxA2: Thromboxane A2
